# Clinical and epidemiological features and prognosis of complicated pyelonephritis: a prospective observational single hospital-based study

**DOI:** 10.1186/s12879-014-0639-4

**Published:** 2014-12-10

**Authors:** Veronica A Buonaiuto, Ignacio Marquez, Inmaculada De Toro, Carolina Joya, Juan D Ruiz-Mesa, Raimundo Seara, Antonio Plata, Beatriz Sobrino, Begoña Palop, Juan D Colmenero

**Affiliations:** Infectious Diseases Department, Regional University Hospital, Málaga, Spain; Microbiology Unit, Regional University Hospital, Malaga, Spain; Critical Care and Emergency Departments, Regional University Hospital, Malaga, Spain; IBIMA, Malaga University, Malaga, Spain

**Keywords:** Urinary tract infection, Complicated, Epidemiology, Prognosis

## Abstract

**Background:**

Complicated pyelonephritis (cPN), a common cause of hospital admission, is still a poorly-understood entity given the difficulty involved in its correct definition. The aim of this study was to analyze the main epidemiological, clinical, and microbiological characteristics of cPN and its prognosis in a large cohort of patients with cPN.

**Methods:**

We conducted a prospective, observational study including 1325 consecutive patients older than 14 years diagnosed with cPN and admitted to a tertiary university hospital between 1997–2013. After analyzing the main demographic, clinical and microbiological data, covariates found to be associated with attributable mortality in univariate analysis were included in a multivariate logistic regression model.

**Results:**

Of the 1325 patients, 689 (52%) were men and 636 (48%) women; median age 63 years, interquartile range [IQR] (46.5-73). Nine hundred and forty patients (70.9%) had functional or structural abnormalities in the urinary tract, 215 (16.2%) were immunocompromised, 152 (11.5%) had undergone a previous urinary tract instrumentation, and 196 (14.8%) had a long-term bladder catheter, nephrostomy tube or ureteral catheter. Urine culture was positive in 813 (67.7%) of the 1251 patients in whom it was done, and in the 1032 patients who had a blood culture, 366 (34%) had bacteraemia. *Escherichia coli* was the causative agent in 615 episodes (67%), *Klebsiella spp* in 73 (7.9%) and *Proteus ssp* in 61 (6.6%). Fourteen point one percent of GNB isolates were ESBL producers. In total, 343 patients (25.9%) developed severe sepsis and 165 (12.5%) septic shock. Crude mortality was 6.5% and attributable mortality was 4.1%. Multivariate analysis showed that an age >75 years (OR 2.77; 95% CI, 1.35-5.68), immunosuppression (OR 3.14; 95% CI, 1.47-6.70), and septic shock (OR 58.49; 95% CI, 26.6-128.5) were independently associated with attributable mortality.

**Conclusions:**

cPN generates a high morbidity and mortality and likely a great consumption of healthcare resources. This study highlights the factors directly associated with mortality, though further studies are needed in the near future aimed at identifying subgroups of low-risk patients susceptible to outpatient management.

## Background

Acute pyelonephritis (aPN) is a severe form of urinary tract infection (UTI) with symptoms that range from mild discomfort to life-threatening illness or death [1],[2]. The incidence of aPN is estimated at around 9–11 cases per 10,000 inhabitants, and it is 4–5 times more frequent in women than in men [3]. Acute pyelonephritis is a common cause of hospital admission and it has a very high economic impact, over 2 billion dollars yearly in the United States [4].

The concept of complicated urinary tract infection (cUTI) encompasses a very heterogeneous group of patients, about whose composition there is no unanimous agreement [5]-[7]. Although much information is available about the epidemiological characteristics and prognosis of uncomplicated aPN [8]-[12], information about the epidemiological, clinical and microbiological characteristics and prognosis of complicated pyelonephritis (cPN) is very scarce [13].

The correct and rapid recognition of cPN is important because when complicating factors are present, antimicrobial resistance is more common and the response to therapy is often disappointing, even with agents active against the pathogen [14]. Although it is generally assumed that all patients with cPN should be admitted, no solid studies have been undertaken to support this [15]. The aim of this study was to describe the epidemiological, clinical and microbiological characteristics and prognosis of cPN in a large, representative sample of patients.

## Methods

### Study design, population and setting

This prospective, single hospital-based observational study included 1325 consecutive inpatients aged 14 years or older with cPN admitted between 1 July 1997 and 30 June 2013 to the University Regional Hospital of Malaga, a 1100-bed tertiary university hospital located in the south of Spain and covering a population of about 350,000 inhabitants.

### Definitions

Acute pyelonephritis was defined as the presence of two of the following: (a) axillary temperature ≥ 38.3°C or chills; (b) flank pain or costovertebral angle tenderness or pain on bimanual palpation of the kidney; and (c) mictional syndrome (including two or more of the following; dysuria, frequency, suprapubic pain or urgency), together with the presence of pyuria (a positive leukocyte esterase dipstick test result, subsequently confirmed by urinalysis with more than 10 leukocytes/mL in urine without centrifuging or more than 5 leukocytes per high-power field in centrifuged sediment) or a positive urine culture.

Complicated PN was defined as PN occurring in any male patient, in patients with functional or anatomical abnormalities of the urinary tract, immunosuppressed persons, patients with a single kidney, permanent bladder catheter, nephrostomy or double-J catheter, or those patients who had experienced urinary tract manipulation in the previous two weeks.

The criteria for systemic inflammatory response syndrome (SIRS), sepsis, severe sepsis and septic shock were established according to the SCCM/ESICM/ACCP/ATS/SIS International Sepsis Definitions Conference [16]. SIRS was defined as the presence of two or more of the following conditions: fever (axillary temperature > 38.3°C) or hypothermia (< 36°C), tachypnoea (> 24 breaths/min), tachycardia (heart rate > 90 beats/min), leukocytosis (> 12,000 cells/mL), leucopoenia (< 4,000 cells/mL), or greater than 10% bands. Patients were considered to have sepsis if they had the criteria for SIRS and a clinical or microbiologically verified infection. Severe sepsis was defined as sepsis associated with hypoperfusion abnormalities such as prerenal azotemia, oliguria, altered mental status, acrocynosis and/or coldness, or hypotension. Septic shock was defined as sepsis with a systolic blood pressure < 90 mm Hg, or 40 mm Hg less than the patient’s baseline blood pressure for at least 1 hour, despite adequate fluid resuscitation

### Exclusion criteria

Patients were excluded from the study if they were in the postoperative period after major urological surgery, had a kidney transplant, or were pregnant.

The data were collected prospectively following a specifically designed protocol. The variables recorded were age, gender, presence of an underlying nephro-urological condition (renal lithiasis, non-lithiasic ureteral obstruction, structural bladder disorder, functional bladder disorder, prostatic disorder, permanent bladder catheter, nephrostomy catheter, non-surgical urological manipulation in the previous two weeks), predisposing factors (including previous history of UTIs, diabetes, active malignancies and chronic renal failure), clinical presentation data, duration of symptoms, previous antimicrobial treatment, and physical examination. Haematological and biochemical parameters, including total leukocyte and differential blood cell counts, haemoglobin, haematocrit and platelet count, creatinine and C-reactive protein levels. Blood and urine cultures were done for most of the patients on admission, as was an abdominal ultrasound either at the time of arrival at the hospital or within 48 hours of admission. Other laboratory and radiographic investigations, with the exception of those required in the research protocol, were conducted at the discretion of each specialist in emergency medicine, internal medicine or infectious diseases.

### Microbiological studies

For blood cultures, 10 mL of venous blood was obtained from all the patients and inoculated into aerobic and anaerobic bottles. At least two sets of blood cultures were obtained from each patient. Blood cultures were processed following conventional protocols for 5 days in a semiautomatic BACTEC 9240 device (Becton Dickinson Diagnostic Instruments System Sparks, MD, USA). The following isolates were considered to be contaminants if they were isolated in only one culture bottle and discordant from urine cultures: *Bacillus* spp, *Corynebacterium* spp, *Micrococcus* spp, and coagulase-negative *staphylococci* except *Staphylococcus saprophyticus.*

Urine specimens for culture were obtained using the midstream clean-catch urine technique or straight catheterization method. In the case of a urinary catheter, the urine sample was collected from the port of the catheter. All urine cultures were inoculated on blood agar and McConkey agar. A positive urine culture was defined as the presence of one or two uropathogens at 10^4^ cfu/mL or greater, or ≥ 10^3^ cfu/mL in the presence of pyuria when the patient had a bladder or nephrostomy catheter. Urine cultures revealing growth of more than two different bacterial species reflecting mixed skin or gut flora were considered to indicate contamination. Positive urine cultures underwent identification and sensitivity analysis in VITEK 2 (bioMerieux Inc, Durham, NC) or WIDER (Soria Melguizo SA). The resistances detected (presence of ESBL, carbapenemases, etc.) were confirmed routinely on disk plate (ROSCO Diagnostica A/S, Taastrup, Denmark) and with selective media (bioMerieux Inc, Durham, NC). All cultures and susceptibility were tested using National Committee for Clinical Laboratory Standards procedures [17]. A discordant culture result was defined as a positive blood culture with a related urine culture that showed growth of another microorganism, did not show bacterial growth, or was contaminated. When the results of the urine and the blood cultures were discordant, the aetiology of the episode of cPN was attributed to the microorganism that produced the bacteraemia.

### Clinical evaluation, antibiotic therapy, and follow-up

After the initial diagnosis all the patients were admitted to a conventional hospital ward or the intensive care unit depending on their clinical status and treated immediately, first empirically and then, after identification of the causative microorganism, with targeted therapy, for not less than 10 days and always in accordance with the Guidelines for Antimicrobial Therapy of the University Regional Hospital of Malaga. An empirical treatment schedule was considered appropriate when it was shown to contain at least one active agent against the causative microorganism of the particular episode of cPN and was used at the recommended dose.

Any alterations on the imaging studies that affected the treatment were considered as clinically relevant urological disorders. The duration of symptoms was defined as the time from the day of presentation to the day of admission. Defervescence was defined as the time needed for the axillary temperature to be less than 37°C in the presence of clinical improvement. Therapeutic failure was considered to be the persistence or worsening of the symptoms after 5 days of adequate treatment. Relapse was defined as the presence or the reappearance of symptoms or signs of the infection with the same microorganism within 4 weeks after discontinuation of the antibiotic treatment and re-infection as recurrence due to a different bacterial strain.

### Statistical analysis

Data were entered into SPSS software version 17.0 (SPSS Inc, Chicago, Ill, USA). Results are expressed as the median and interquartile range (IQR), or number and percentage. Normally and non-normally distributed quantitative variables were compared using *t*-tests and Mann–Whitney U-tests respectively. Categorical variables were compared using the χ2 test or Fisher exact test when appropriate. Measures for association were expressed as odds ratios (OR) with their 95% confidence intervals (CI) for dichotomous variables. A 2-tailed *P* value of 0.05 was considered statistically significant. Covariates found to be associated with attributable mortality on univariate analysis at a level of significance P < 0.1 were eligible for inclusion in a multivariate logistic regression model using a backward selection procedure.

The study was approved by the Ethics Committee of the University Regional Hospital of Malaga, which waived the need for informed consent, as all the data and samples were collected as part of normal care in daily clinical practice, according to the current guidelines.

## Results

Of the 1325 patients included in the study, 689 (52%) were men and 636 (48%) women. The median age was 63 years, (IQR 46.5-73). Overall, 1116 (84.2%) patients were admitted to the Infectious Diseases or Internal Medicine Departments, 198 (14.9%) required admission to the Intensive Care Unit, and 11 (0.9%) were admitted to other medical wards. It was the first episode of cPN in 830 (62.6%) patients; the remaining 495 (37.3%) had experienced previous episodes.

Most episodes (940; 70.9%) occurred in patients with structural or functional abnormalities of the genitourinary tract. Table 1 shows the factors based on which the patients were considered to have cPN. Recurrent lower UTI was reported by 309 (23.3%) patients and 570 (43%) had other disorders, such as diabetes (382; 28.8%) or chronic kidney failure (96; 7.2%).

**Table 1:** **Criteria categorizing patients with complicated pyelonephritis**

	N° (%) of patients		
	Males	Females	Overall
Urolithiasis	112 (16.2)	226 (35.5)	338 (25.5)
Neurogenic bladder	118 (17.1)	89 (14.0)	207 (15.6)
Benign prostatic hyperplasia	203 (29.5)	0	203 (15.3)
Structural bladder pathology	76 (13.0)	96 (15.1)	172 (13)
Urinary tract instrumentation	105 (15.2)	47 (7.4)	152 (11.5)
Long-term bladder catheterization	95 (13.8)	32 (5.0)	127 (9.6)
Non lithiasis ureteral or urethral obstruction	52 (7.7)	55 (8.6)	107 (8.1)
Anatomical or functional single kidney	24 (3.5)	33 (5.2)	57 (4.3)
Nephrostomy tube	26 (3.8)	28 (4.4)	54 (4.1)
Ureter catheter or stent	8 (1.2)	7 (1.1)	15 (1.1)
Immunocompromised	92 (13.5)	123 (19.3)	215 (16.2)

The median duration of symptoms prior to admission was 3.0 days (IQR 2–6). Most (87%) of the patients had fever ≥ 38.3°C, 59.4% had flank pain, and 56.7% costovertebral tenderness. The main clinical, haematological, biochemical and urinalysis data are shown in Table 2. Serum C-reactive protein concentrations were available for 625 patients, in whom it was elevated in 587 (93.9%), with an overall median of 143 mg/L (IQR 65.5-224).

**Table 2:** **Main clinical, laboratory and microbiological data**

	Number of cases (%)
Fever ≥ 38°C	1155 (87.2)
Chills	982 (74.1)
Flank pain	789 (59.5)
Costovertebral tenderness	751 (56.7)
Mictional syndrome	817 (61.6)
Systolic blood pressure ≤ 90 mm Hg	266 (20.2)
Leukocytosis	989 (74.7)
Thrombopoenia	190 (14.3)
C-reactive protein ≥ 6 mg/L^a^	587 (93.9)
Pyuria	1164 (87.9)
Microscopic haematuria	527 (39.8)
Bacteriuria	382 (28.8)
Pathogens responsible for episodes of Complicated pyelonephritis	
*Escherichia coli*	615 (67)
*Klebsiella spp*	73 (7.9)
*Proteus spp*	61 (6.1)
*Enterobacter spp*	33 (3.6)
Non-fermenting GNB^b^	50 (5.4)
*Enterococcus faecalis*	31 (3.4)
Other Gram positive bacilli	8 (0.9)
Polymicrobial	23 (2.5)
*Candida spp*	10 (1.1)
Others	14 (1.5)

^a^The data refer to 625 patients.

^b^Gram negative bacilli.

In the week prior to admission 508 patients (38.4%) had received some antimicrobial treatment. A urine culture was performed for 1251 patients and blood culture for 1076. The urine culture was positive in 813 (67.7%) patients and 378 (35.1%) patients had a clinically relevant bacteraemia. Both urine and blood cultures were done for 1032 (77.9%) patients, with 126 (12.2%) discordant results; 35 (27.7%) due to a different isolate and 91 (72.3%) due to isolation of a uropathogen in the blood cultures even though the urine culture was negative.

*Escherichia coli* was the causative agent in 615 (67%) episodes, *Klebsiella spp* in 73 (7.9%) and *Proteus spp* in 61 (6.6%). Table 2 also shows the aetiological agents in the 918 (69.3%) patients in whom the aetiology of the cPN was eventually confirmed. Of GNB isolates 14.1% were ESBL producers; 13.8% for *E. coli* and 17.7% in the case of *Klebsiella spp.*

On arrival at the hospital or within 48 hours of admission, an ultrasonographic study was available in 1109 cases. Of these, ultrasonography showed some pathological finding in 651 cases (58.7%). A CT was done for 279 patients (21.1%), in most cases within 5 days of hospital admission. CT was pathological in 223 (79.9%) cases. The ultrasound findings were considered very relevant in 80 (7.27%) cases, relevant in 402 (36.2%), and incidental in 28 (2.5%). Table 3 shows the most clinically relevant CT and ultrasound findings. In 23 (57.5%) of the 40 patients who were shown to have abscesses the diameter of these was greater than 3 cm. All 23 patients underwent drainage of the collection using ultrasound- or CT-guided puncture.

**Table 3:** **Main imaging findings in patients with complicated pyelonephritis**

	Ultrasonography Number = 1109	CT Number = 279
	N° (%) of patients	
***Clinically relevant finding***	482 (43.7)	223 (79.9%)
Ectasia ≥ grade II	203 (18.3)	124 (44.4)
Kidney or ureteral stones	199 (17.9)	24 (8.6)
Complicated kidney cyst	83 (7.5)	26 (9.3)
Kidney or perinephric abscess	25 (2.6)	27 (9.7)
Focal nephritis	15 (1.4)	42 (15.1)
Others^a,b^	8 (0.7)^a^	27 (9.7)^b^

^a^Four patients with findings suggestive of kidney neoplasia, 2 with prostatic mass and one with psoas abscess and prostatic abscess respectively. ^b^Thirteen patients with findings suggestive of kidney neoplasia, 8 with psoas abscess and 6 with prostatic abscess.

The initial empirical treatment was combined therapy in 23.6% of the cases and single therapy in 76.4%, being considered appropriate in 96.6% of the cases in which the aetiology was confirmed. Severe sepsis occurred in 343 (25.9%) patients and septic shock in 165 (12.5%). The median number of days before reaching apyrexia was 2 (IQR 1–3) and the median hospital stay was 9 days (IQR 6–14). During their admission 87 patients (6.6%) died, with the cause of death being directly related with the episode of cPN in 54 (4.1%). After hospital discharge 21 patients (1.7%) failed to attend for review. Of the remaining 1217, 1066 (87.6%) were cured, 98 (8.0%) had a relapse, and 53 (4.3%) experienced a reinfection.

In univariate analysis, an age > 75 years, immunosuppression, chronic kidney failure, the absence of fever or costovertebral tenderness, systolic blood pressure < 90 mm Hg, high peripheral blood white cell count, thrombocytopoenia, creatinine levels > 2 mg/%, bacteraemia, the presence of severe sepsis, acute renal failure and septic shock were significantly associated with attributable mortality. After multivariate analysis, only an age > 75 years, immunosuppression and septic shock were associated independently with attributable mortality (Table 4).

**Table 4:** **Risk factors associated with attributable mortality in patients with complicated pyelonephritis**

	Univariate	*p*	Multivariate
	OR (95% CI)		OR (95% CI)
Age > 75 years	1.05 (1.01-1.19)	.002	2.77 (1.35-5.68)
Long-term bladder catheterization	1.97 (0.94-4.14)	.091	
Immunosuppression	2.81 (1.56-5.05)	.001	3.14 (1.47-6.70)
Chronic kidney failure	2.37 (1.16-4.85)	.021	
Absence of fever	1.05 (1.01-1.07)	.001	
Absence of costovertebral tenderness	1.04 (1.02-1.70)	.005	
Systolic blood pressure <90 mm Hg	3.90 (2.24-6.80)	.001	
Leukocytosis > 20.000 cell/ml	1.03 (1.00-1.06)	.031	
Thrombocytopoenia	2.72 (1.46-5.05)	.009	
Serum creatinine > 2 mg /dL	1.10 (1.05-1.15)	.001	
Ectasia ≥ grade II	1.99 (0.99-3.99)	.059	
Bacteraemia	2.32 (1.20-4.48)	.009	
Acute kidney failure	7.56 (4.01-14.30)	.001	
Severe sepsis	17.33, (8.37-35.89)	.001	
Septic shock	54.5 (25.9-114.5)	.001	58.5 (26.6-128.5)

## Discussion

Symptomatic infection in patients with cUTI varies across a wide spectrum, ranging from mild lower tract symptoms to aPN with systemic manifestations such as bacteraemia and severe sepsis.

Independently of whether cPN is community acquired, associated with health care or nosocomial, its incidence is still high [18],[19]. The little information available about its clinical and microbiological characteristics, as well as its prognosis, is therefore surprising. Multiple explanations exist for this apparent paradox, though the main one relates to the great difficulty to undertake systematic studies given the lack of consensus concerning the correct definition of cPN. This is why cPN has been excluded from the guideline recommendations [20]. Additionally, cPN is either expressly excluded in most clinical trials in UTI [21],[22], or is assessed together with a heterogeneous mixture of complicated lower UTI, aPN and cPN [23],[24].

Some studies exist on cPN in specific subgroups of patients [25],[26] but to our knowledge the study reported here is the largest prospective study of patients with acute cPN, in which patients were selected and treated on the basis of well-defined and homogeneous criteria. Our findings show that in cPN the proportion of men is similar to that of women. This does not appear to have been influenced by the fact that all the cases of pyelonephritis in men were considered complicated, as in our study over 90% of the men had other inclusion criteria as well. Thus, in concordance with other studies, our results show the current tendency for the need to consider all cases of PN in men as complicated [5],[6],[11].

The median duration of the symptoms prior to admission was 3 days, a figure similar to that reported in a recent study [27].

The yield of the urine culture was 67.7%, a lower figure than that for uncomplicated PN, though very similar to that reported in recent studies that just included patients with cPN [28]. In addition, in our study 34% of the patients had clinically relevant bacteraemia, a figure similar to the 35.6%-42% reported in other studies of cPN [13],[28].

The discordance between the results of the urine and blood cultures in uncomplicated PN does not usually exceed 2-3%, which explains why some authors advocate just a urine culture in these cases [29],[30]. On the other hand, a discordance between urine and blood cultures in cPN is not unusual. In our study the percentage of discordance in the patients for whom both a urine and a blood culture were available was 12.2%, and in over two thirds of cases this discordance was due to the presence of bacteraemia in a patient with a negative urine culture. Similar findings have been reported before [31]. During the week prior to arrival at the hospital 38.5% of the patients included in our study had received some antibiotic therapy. This circumstance, very common in clinical practice and mentioned in previous studies, partly explains the yield of the urine culture [31]. The second reason for the discordance could lie in the fact that an important percentage of patients who come clearly septic to the emergency areas have blood cultures drawn immediately, after which antimicrobial therapy is initiated empirically with a delay in the urine culture. This procedure explains in great part the discordance due to the presence of bacteraemia but a negative urine culture.

Although *Escherichia coli* is the most frequent aetiological agent in any ITU, in cPN the frequency of its isolation does not exceed 65-70% (67% in our study) whereas more importance is assumed by other fermenting GNB, like *Klebsiella spp* or *Proteus ssp,* or non-fermenting GNB, like *Pseudomonas aeruginosa* or *Acinetobacter baumanii*. A recent study including 800 patients with cUTI, most of which concerned cPN, found that *Escherichia coli* was responsible for 63% of the episodes and *Klebsiella spp* for 7% [31]. In our study the proportion of ESBL producing microorganisms was 14.1%, slightly higher than that reported by other authors [32],[33]. This aetiological spectrum and the ever increasing proportion of ESBL producing and multiresistant microorganisms demand a special effort in the aetiological diagnosis of cPN.

Because ultrasonography is a non-invasive, radiation-free, very versatile, widely available and highly efficient method, it is the imaging technique of choice for any patient with nephro-urologic pathology. However, the contribution of ultrasonography to the decision-taking process in aPN is limited, which explains why many authors consider it unnecessary in this clinical setting when the patient progresses satisfactorily with adequate treatment, as is usually the case [34],[35]. Notwithstanding this, the role of ultrasonography in the initial management of cPN has been poorly studied, which explains the lack of a wide consensus for this aspect. In our study an ultrasound examination was available for 83.7% of the patients either on arrival at the hospital or within 48 hours of admission, detecting clinically relevant urological disorders in 43.5% of examinations. A previous prospective study involving 245 patients with febrile UTI found that ultrasonography detected 19.2% of clinically relevant urological disorders. This figure is not very different to that in our study bearing in mind that 40% of their patients were diagnosed with aPN [36]. Although these authors propose a prediction rule to select the patients with PN who could benefit from an ultrasound study, we believe that, given the high morbidity and high prevalence of alterations requiring urgent instrumentation, an ultrasonographic study should be performed in all cPN patients.

It is well known that CT is superior to ultrasonography in urologic disorders [37]. Indeed, in our study CT provided relevant findings in 79% of cases in which it was performed. However, since it requires potentially nephrotoxic contrast enhancement, as in our study, CT should be reserved for those patients who progress unfavourably and whose prior ultrasound studies are either normal or inconclusive.

The prognosis for patients with cPN is much worse than for those with aPN. The information available to date about the prognosis for cPN and its influencing factors is scarce and limited to a few very particular settings [28]. In our study 26% of the patients developed severe sepsis and 12.5% septic shock, figures that are very similar to those reported in studies that only included patients with cPN [15],[27]. The percentage of patients with septic shock may in fact be even higher, reaching 21% in patients with obstructive uropathy [28]. The severity of the clinical picture explains why a high percentage of patients require admission to Intensive Care Units, which was the case for 15% of the patients in our study.

Mortality associated with cPN ranges between 3.2% and 9.8% [11],[38],[39]. In our study the crude mortality was 6.5% and the attributable mortality was 4.1%. To the best of our knowledge no other study has analyzed crude and attributable mortality separately. This difference is very relevant because a high percentage of patients with cPN have important comorbidities that can contribute directly to mortality. Therefore, when we analyzed the factors related with mortality in our study we only included those cases whose mortality was attributable exclusively to the PN. The factors identified that were more closely related with mortality were an age over 75 years, a state of immunosuppression, chronic renal failure, a permanent bladder catheter, a systolic blood pressure less than 90 mm Hg, leukocyte figures above 20,000 cells per mL, thrombopoenia, the presence of bacteraemia, severe sepsis, and septic shock. Most of these factors have been previously identified in various studies as factors of poor prognosis [11],[13],[15],[27],[28],[38],[40]. A few authors have related the presence of bacteraemia with a worse prognosis in patients with cPN [28], though this was not confirmed in our study nor has it been confirmed in other studies [11],[27],[40]. In fact, bacteraemia is very common in aPN and most of these patients are managed as outpatients, with an excellent prognosis [30].

In our study only an age over 75 years, immunosuppression, and the development of septic shock were independently associated with a greater attributable mortality. These factors, to which should be added a bedridden status (not included in our study), have also been identified in previous studies [15],[27],[28]. It is well known that older patients, debilitated patients, or those with comorbidities often have a poor performance status and present a less expressive clinical picture, which can delay diagnosis and the corresponding initiation of adequate therapy.

This study suffers from a few limitations. First, it did not include postoperative patients after major urological surgery or kidney transplant patients, both groups that clearly fulfil criteria for cPN. The main reason for their exclusion was that these two groups of patients represent very particular situations, with such a specific epidemiology that they might not be representative of most patients, who usually have a community-acquired infection, are initially managed by primary care physicians, and who often attend the emergency areas. Second, our study did not consider complicated those cases of pyelonephritis occurring in postmenopausal women or those in diabetic patients just because they were diabetic. These two situations, though, are not currently considered by most authors as risk factors for cUTI [20].

Nevertheless, the study has important strengths, as it concerns the largest sample of cPN patients reported to date. All the patients were recruited based on well-defined criteria and managed homogenously at a single hospital, circumstances that presumably confer a high degree of external validity to the results.

## Conclusion

In conclusion, cPN is a very prevalent infection that involves a high consumption of healthcare resources, and often has a very severe clinical course with considerable mortality, particularly among patients who are older, immunosuppressed, or who develop septic shock. Although cPN is currently considered a disorder with a clear indication for hospital admission, there may exist subgroups of patients who can safely be managed as outpatients. Further studies are desirable aimed at answering this question.

## Abbreviations

aPN: Acute pyelonephritis
UTI: Urinary tract infection
cUTI: Complicated urinary tract infection
cPN: Complicated pyelonephritis
IQR: Interquartile range
GNB: Gram negative bacilli
ESBL: Extended spectrum betalactamase
